# RefineSleepNet: Task-Driven Signal-Domain Transformation for EEG/EOG Sleep Staging

**DOI:** 10.3390/bioengineering13101146

**Published:** 2026-09-30

**Authors:** Zhaoyan Zhu, Bo Yin, Pengyu Jiang, Yue-Ting Sun

**Affiliations:** College of Information Science and Engineering, Ocean University of China, Qingdao 266100, China; zzy_ouc@stu.ouc.edu.cn (Z.Z.); 11250211007@stu.ouc.edu.cn (P.J.); syt21997@163.com (Y.-T.S.)

**Keywords:** automatic sleep staging, EEG/EOG, signal-domain transformation, multimodal fusion, temporal modeling

## Abstract

Automatic sleep staging relies on effective representation of physiological signals. Representative raw-signal sleep-staging encoders typically transform input waveforms while progressively compressing their temporal resolution within the same encoding path. However, explicit modality-specific, sample-aligned, and task-driven signal-domain transformation before epoch-level feature extraction remains comparatively underexplored. To investigate this design, we propose RefineSleepNet, an end-to-end sequence-to-sequence framework that explicitly separates these two stages. Separately parameterized Signal Refinement U-Net (SRU-Net) branches first transform EEG and EOG waveforms while preserving the original temporal length, after which Epoch Feature Extractors (EFEs) extract epoch-level representations from the refined signals. Fused Sequence Attention (FSA) then performs multimodal integration and cross-epoch contextual modeling before epoch-wise classification. All stages are jointly optimized using the final sleep-staging objective without a separate waveform reconstruction target. RefineSleepNet was evaluated on Sleep-EDF-39, Sleep-EDF-153, and ISRUC-3 using subject-independent 10-fold cross-validation, achieving accuracy/macro-F1 (MF1) scores of 84.5%/79.8%, 83.5%/78.1%, and 80.3%/78.1%, respectively. Ablation experiments supported the coordinated design, while statistical analysis and intermediate-representation evaluations further examined the robustness and characteristics of the learned transformation. The learned transformation retained broad temporal and spectral organization while introducing local waveform changes and modality-specific spectral redistribution. These results support the staged organization as a viable modeling strategy for EEG/EOG sleep staging.

## 1. Introduction

Sleep is a fundamental physiological process that contributes to neural information processing and memory-related organization [1]. Sleep staging provides a standardized description of overnight sleep organization by segmenting polysomnography (PSG) recordings into 30 s epochs and assigning each epoch to one of five stages: Wake (W), N1, N2, N3, and rapid eye movement (REM), according to the American Academy of Sleep Medicine (AASM) criteria [2]. Among PSG signals, electroencephalogram (EEG) and electrooculogram (EOG) are widely used because they provide complementary cortical and ocular information associated with sleep-stage transitions. However, manual sleep staging remains time-consuming, labor-intensive, and subject to inter-scorer variability. Therefore, automatic sleep staging has become an important approach for efficient analysis of overnight PSG recordings.

Early automatic sleep staging methods mainly relied on handcrafted time-domain, frequency-domain, and time–frequency features combined with conventional classifiers [3]. Although these approaches demonstrated the feasibility of automatic sleep staging, their performance depended strongly on manually designed feature representations. Deep learning methods subsequently reduced this dependence by learning sleep-related representations directly from raw or transformed signals. CNN-based approaches have been widely adopted to capture local and multiscale waveform patterns within individual epochs [4,5], while dual-view architectures further exploit complementary amplitude and temporal characteristics of EEG signals [6]. Fully convolutional encoder–decoder frameworks have also been introduced for dense temporal sleep-stage classification, such as U-Time, which performs end-to-end segmentation using an encoder–decoder architecture [7]. Although these approaches have achieved promising performance, most studies primarily focus on extracting discriminative representations from input signals, whereas the organization of signal transformation and subsequent representation compression remains less explicitly investigated.

Beyond epoch-level feature learning, sleep staging also requires modeling temporal dependencies across neighboring epochs because sleep stages evolve sequentially rather than independently. Existing studies have introduced recurrent architectures [8,9,10,11,12], attention-based approaches [13,14,15], and more recent Transformer-based or hybrid frameworks [16,17,18,19]. These developments have substantially improved the ability to capture cross-epoch contextual information. However, most sequence modeling methods operate on already extracted epoch-level features, and the interaction between signal-domain transformation and subsequent temporal dependency modeling remains insufficiently explored.

In parallel, multimodal approaches have leveraged complementary information from EEG, EOG, and other PSG signals to improve sleep-stage representation. Existing studies have investigated joint-input, feature-level fusion, multi-view learning, ensemble strategies, and learned fusion mechanisms [20,21,22,23,24]. These studies demonstrate the value of multimodal physiological information for automatic sleep staging. Nevertheless, EEG and EOG reflect different physiological processes, and many existing approaches mainly combine these modalities after feature extraction, leaving modality-specific signal refinement before fusion relatively underexplored.

Despite these advances, several challenges remain in learning robust representations for EEG/EOG-based sleep staging. First, in many raw-signal sleep-staging frameworks, waveform transformation and temporal compression are performed within the same encoding pathway. Although effective for classification, such coupled designs make it difficult to explicitly characterize how physiological signals are transformed before epoch-level representation learning. Second, EEG and EOG contain complementary but distinct information, while existing multimodal frameworks provide limited investigation into modality-specific signal-domain transformation before fusion. Third, although cross-epoch modeling has improved sleep-stage transition recognition, how to effectively integrate temporally aligned signal representations with sequence-level contextual modeling remains an open problem.

To address these challenges, this study investigates a staged representation-learning architecture in which modality-specific, sample-aligned signal-domain transformation is explicitly performed before epoch-level feature compression and sequence-level contextual modeling. Since EEG and EOG exhibit distinct signal characteristics while providing complementary sleep-stage information, separately parameterized transformation branches are designed for the two modalities. After signal refinement and epoch-level feature extraction, the resulting modality-specific representations are integrated and modeled across consecutive epochs to incorporate multimodal complementarity and temporal dependencies.

To implement this staged architecture, we propose RefineSleepNet, an end-to-end sequence-to-sequence framework for EEG/EOG sleep staging. For each epoch, separately parameterized Signal Refinement U-Net (SRU-Net) branches first generate modality-specific, sample-aligned signal representations while preserving the original temporal length. Epoch Feature Extractors (EFEs) then compress these representations into fixed-dimensional epoch-level features. The resulting EEG and EOG feature sequences are integrated and contextually modeled across consecutive epochs by the Fused Sequence Attention (FSA) module, followed by epoch-wise classification. The entire framework is optimized jointly using the final sleep-staging objective without requiring an explicit waveform reconstruction or denoising target.

The main contributions of this work are summarized as follows:

1. We investigate a staged representation-learning strategy that explicitly decouples modality-specific, sample-aligned signal-domain transformation from subsequent epoch-level feature compression. The transformation preserves the original temporal length and is optimized jointly through the final sleep-staging objective rather than a predefined waveform reconstruction target.
2. We develop RefineSleepNet to instantiate this strategy for multimodal EEG/EOG sleep staging. Separately parameterized SRU-Net branches learn modality-specific signal representations, followed by epoch-level feature extraction, multimodal feature integration, and cross-epoch contextual modeling within a unified sequence-to-sequence framework.
3. We conduct systematic evaluations on three public sleep datasets using subject-independent cross-validation, representative baseline comparisons, statistical analyses, and controlled ablation studies. Beyond classification performance, the learned intermediate representations and predicted sleep architecture are investigated through temporal consistency, spectral modulation, intra-epoch attention-response analysis, and agreement analysis between predicted and reference hypnograms.

## 2. Materials and Methods

### 2.1. Datasets and Preprocessing

Three public sleep datasets, including Sleep-EDF-39, Sleep-EDF-153, and ISRUC-3 [25,26,27], were used to evaluate the proposed RefineSleepNet framework. Sleep-EDF-39 comprises 20 healthy subjects, including 10 males and 10 females aged 25 to 34 years, yielding 42,308 epochs; except for subject 13 with one missing sleep record, the subjects each have two PSG recordings. Sleep-EDF-153 comprises 78 healthy subjects aged 25 to 101 years with 195,479 epochs; except for subjects 13, 36, and 52, each missing one sleep record, the remaining subjects each have two PSG recordings. ISRUC is a public sleep database with three subgroups, and Subgroup III provides PSG recordings from 10 healthy subjects, resulting in 8785 epochs. Table 1 summarizes the stage distributions of these datasets.

For consistency across datasets, the same preprocessing procedure was applied. Specifically, each 30 s epoch was originally annotated into one of six stages: Wake, N1, N2, N3, N4, and REM. Following the AASM standard, N3 and N4 were merged into a single stage N3 [28]. In addition, as recommended in prior work, we retained the 30 min before sleep onset and the 30 min after the end of sleep. For Sleep-EDF-39 and Sleep-EDF-153, the Fpz–Cz EEG channel and the ROC–LOC EOG derivation were used. For ISRUC-3, the F3–A2 EEG channel and the ROC–A1 EOG derivation were used. All signals were resampled to 100 Hz. No additional signal filtering or normalization was applied. Consecutive 30 s epochs were organized into sequences of 20 epochs, corresponding to a 10 min temporal context. For the training set, a sliding window with a stride of 10 epochs was used, resulting in a 50% overlap between adjacent sequences. Validation and test sequences were constructed without overlap. At the end of each recording, trailing segments containing fewer than 20 epochs were discarded.

### 2.2. Problem Formulation

Automatic sleep staging is formulated as a sequence-to-sequence classification problem, where a consecutive sequence of PSG epochs is used as input and each epoch within the sequence is assigned a corresponding sleep stage label. Unlike independent epoch-wise classification, this formulation allows the model to exploit contextual information from neighboring epochs, which is important because sleep stages exhibit temporal continuity and transition patterns.

Given paired EEG and EOG recordings, the input sequence can be represented as:

(1) $$X^{EEG}=\left\{x_{1}^{EEG},x_{2}^{EEG},\ldots ,x_{T}^{EEG}\right\},$$

(2) $$X^{EOG}=\left\{x_{1}^{EOG},x_{2}^{EOG},\ldots ,x_{T}^{EOG}\right\},$$

where *T* denotes the number of consecutive epochs in an input sequence, and $x_{t}^{EEG}$ and $x_{t}^{EOG}$ represent the EEG and EOG signals of the *t*-th epoch, respectively.

The objective is to learn a mapping function that predicts the sleep-stage sequence:

(3) $$\hat{Y}=F\left(X^{EEG},X^{EOG};\theta \right),$$

where $\hat{Y}=\left\{{\hat{y}}_{1},{\hat{y}}_{2},\ldots ,{\hat{y}}_{T}\right\}$ denotes the predicted sleep-stage labels and θ represents the trainable parameters of RefineSleepNet.

### 2.3. Overview

**Figure 1:**
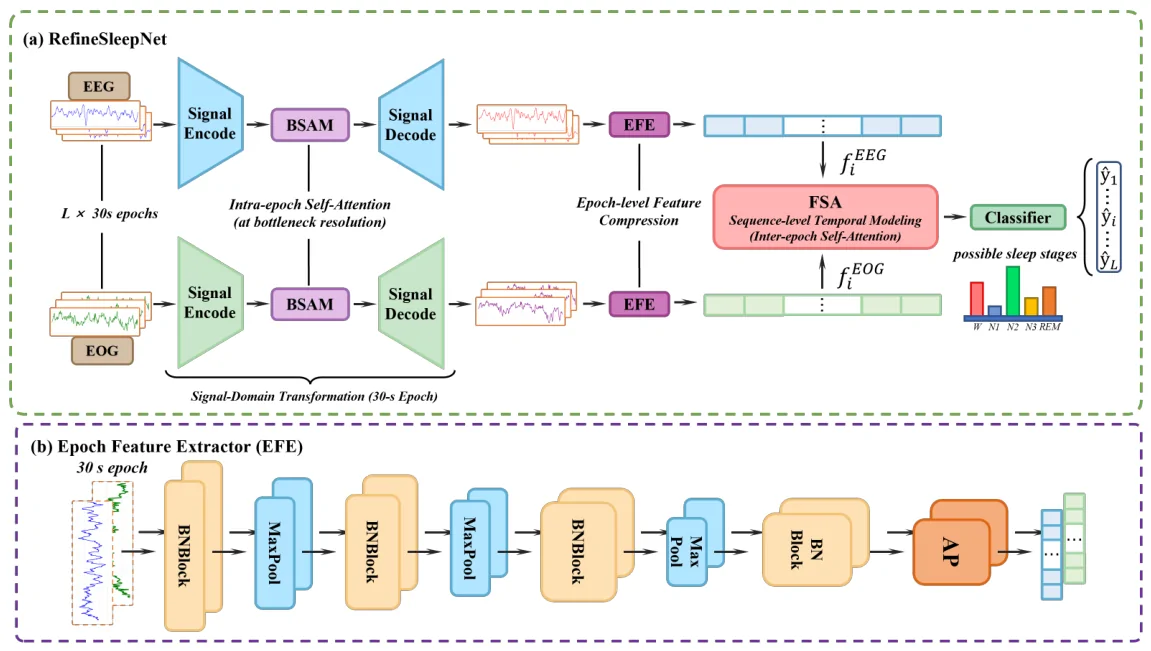
Architecture of RefineSleepNet. (**a**) Overall sequence-to-sequence framework. Consecutive EEG and EOG epochs are first processed by two separately parameterized signal transformation branches, each consisting of a signal encoder, a Bottleneck Self-Attention Module (BSAM), and a signal decoder. The transformed waveforms are then compressed by the Epoch Feature Extractor (EFE) into modality-specific epoch-level feature sequences. The Fused Sequence Attention (FSA) module integrates the EEG and EOG feature sequences and performs cross-epoch contextual modeling, after which a classifier produces one sleep-stage prediction for each input epoch. (**b**) Architecture of the EFE, which consists of successive BNBlocks and temporal max-pooling operations followed by adaptive average pooling.

As illustrated in Figure 1, RefineSleepNet is an end-to-end sequence-to-sequence architecture that organizes representation learning into three stages: modality-specific signal-domain transformation, epoch-level feature extraction, and multimodal sequence modeling. This staged design explicitly separates signal transformation from subsequent feature compression, addressing the limitation of conventional raw-signal encoders where these two operations are often coupled. EEG and EOG are processed independently during the first two stages to preserve modality-specific characteristics, while multimodal interaction and cross-epoch contextual modeling are performed after modality-specific epoch representations have been obtained.

For the *i*-th epoch of modality m∈{EEG,EOG}, the Signal Refinement U-Net (SRU-Net) maps the input signal $x_{i}^{m}\in R^{1\times N}$ to a sample-aligned intermediate representation ${\tilde{x}}_{i}^{m}=R_{m}\left(x_{i}^{m}\right)\in R^{1\times N}$. The Epoch Feature Extractor (EFE) then converts this sample-aligned intermediate representation into a compact epoch-level feature vector for subsequent sequence modeling. The EEG and EOG branches share the same architectural configuration but use separate parameters. Both SRU-Net and EFE operate independently on individual epochs and therefore introduce no cross-epoch interaction.

The resulting sequence representation is finally mapped to one sleep-stage prediction for each input epoch by the classification head. The complete framework is optimized jointly using the final sleep-staging objective, without an explicit waveform reconstruction target, allowing the intermediate signal representation to be learned according to the downstream sleep-staging task rather than a predefined reconstruction criterion.

### 2.4. Signal Refinement U-Net (SRU-Net)

**Figure 2:**
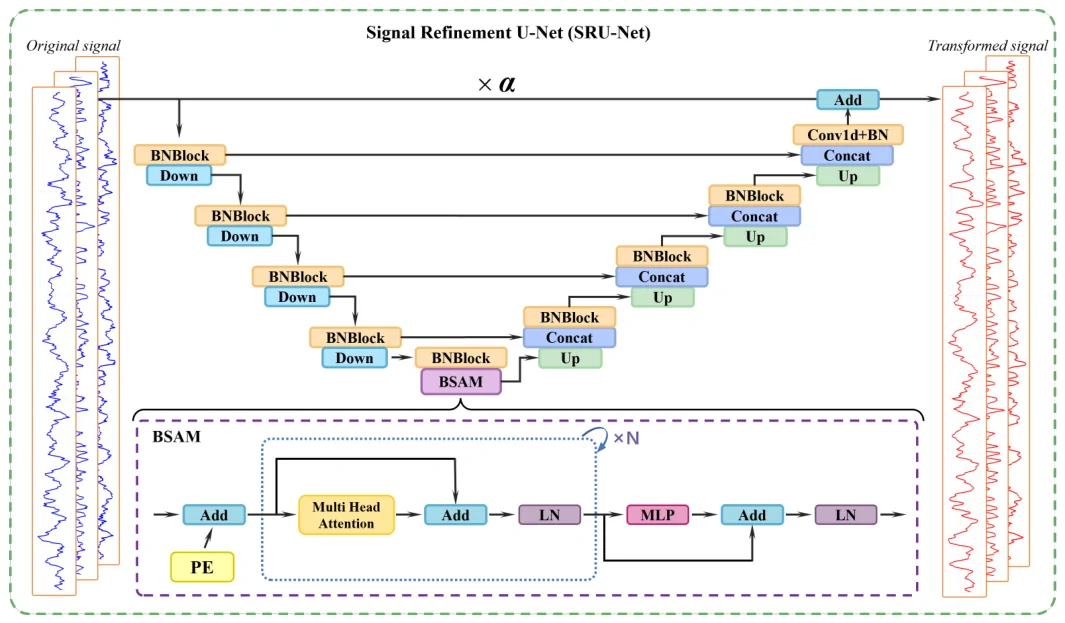
Architecture of a Signal Refinement U-Net (SRU-Net) branch. The encoder extracts multi-scale representations through convolutional blocks and temporal downsampling. At the bottleneck, BSAM applies positional encoding, stacked multi-head self-attention, and a feed-forward network to the compressed temporal representation. The decoder restores the original temporal resolution through upsampling and channel-wise concatenation with the corresponding encoder features. Finally, the decoder output is combined with the original input through a learnable scaled residual shortcut, yielding a sample-aligned intermediate waveform representation.

As illustrated in Figure 2, SRU-Net implements the signal-domain transformation stage before epoch-level feature extraction. For each 30 s epoch, it learns a task-driven signal-domain representation while preserving the original temporal length and sample alignment. EEG and EOG are processed by architecturally identical but separately parameterized SRU-Net branches, allowing modality-specific transformations to be learned without parameter sharing.

For the *i*-th epoch of modality m∈{EEG,EOG}, let $x_{i}^{m}\in R^{1\times N}$ denote the input signal. An initial channel projection is followed by a multi-level encoder composed of convolutional blocks and temporal downsampling:

(4) $$h_{i,0}^{m}=P^{m}\left(x_{i}^{m}\right),\;e_{i,r}^{m}=B_{r}^{m}\left(h_{i,r-1}^{m}\right),\;h_{i,r}^{m}={\mathrm{Pool}}_{r}\left(e_{i,r}^{m}\right),$$

where r=1,…,D, and *D* is the number of encoder stages. $P^{m}$ denotes the initial channel projection, $B_{r}^{m}$ represents the convolution–batch-normalization–activation block at encoder stage *r*, and $e_{i,r}^{m}$ is retained for the corresponding decoder skip connection.

At the bottleneck, the Bottleneck Self-Attention Module (BSAM) is introduced to model dependencies across the compressed temporal positions within each epoch. After bottleneck feature transformation, the channel and temporal axes are transposed, and sinusoidal positional encoding is added:

(5) $$z_{i,0}^{m}={\left[B_{b}^{m}\left(h_{i,D}^{m}\right)\right]}^{⊤}+\mathrm{PE},$$

where $B_{b}^{m}$ denotes the bottleneck convolutional block and PE denotes the sinusoidal positional encoding.

The resulting representation is processed by stacked multi-head self-attention layers. For compactness, ${\mathrm{MHA}}_{a}^{m}\left(z\right)$ denotes self-attention in which the query, key, and value are all derived from z. Each attention layer is formulated as

(6) $$z_{i,a}^{m}={\mathrm{LN}}_{a}^{m}\left(z_{i,a-1}^{m}+{\mathrm{MHA}}_{a}^{m}\left(z_{i,a-1}^{m}\right)\right),$$

where a=1,…,A, and *A* is the number of self-attention layers. The attention stack is followed by a feed-forward network:

(7) $$z_{i,\mathrm{out}}^{m}={\mathrm{LN}}_{f}^{m}\left(z_{i,A}^{m}+{\mathrm{FFN}}^{m}\left(z_{i,A}^{m}\right)\right),$$

where MHA(·), FFN(·), and LN(·) denote multi-head self-attention, the feed-forward network, and layer normalization, respectively. BSAM operates independently within each epoch and therefore captures intra-epoch temporal dependencies. Interactions across consecutive epochs are modeled subsequently by FSA.

The decoder progressively restores the temporal length of the representation. At each decoding stage, the decoder feature is linearly interpolated to the temporal length of the corresponding encoder feature and concatenated with it along the channel dimension:

(8) $$\begin{array}{cc} d_{i,j}^{m}=D_{j}^{m}(\mathrm{Concat} & [{\mathrm{Interp}}_{D-j+1}\left(d_{i,j-1}^{m}\right),\\ \; & e_{i,D-j+1}^{m}]), \end{array}$$

where j=1,…,D, $d_{i,0}^{m}={\left(z_{i,\mathrm{out}}^{m}\right)}^{⊤}$, and $D_{j}^{m}$ denotes the transformation at decoder stage *j*. Moreover, ${\mathrm{Interp}}_{D-j+1}\left(\cdot \right)$ denotes linear interpolation to the temporal length of the corresponding encoder feature, and Concat[·] denotes channel-wise concatenation. The final decoding stage produces a single-channel output with the same temporal length as the original input.

The decoder output is combined with the original waveform through a modality-specific adaptive residual shortcut:

(9) $${\tilde{x}}_{i}^{m}=d_{i,D}^{m}+\alpha_{m}x_{i}^{m},$$

where $\alpha_{m}$ is a learnable scalar initialized to 0.5. This formulation provides an adaptive residual pathway that balances the original signal information and the learned representation, allowing the contribution of the transformed signal to be optimized jointly with the downstream sleep-staging objective. No separate reconstruction or denoising target is imposed; instead, the two SRU-Net branches are optimized jointly with the subsequent staging network using the final sleep-stage classification objective.

### 2.5. Epoch Feature Extractor (EFE)

As shown in Figure 1b, the Epoch Feature Extractor (EFE) converts the sample-aligned output of SRU-Net into a compact epoch-level representation. While SRU-Net produces an output with the same temporal length as its input, EFE progressively compresses the temporal dimension to obtain fixed-dimensional epoch-level features. The EEG and EOG streams employ the same EFE architecture but use separately learned parameters.

For the *i*-th epoch of modality m∈{EEG,EOG}, EFE takes ${\tilde{x}}_{i}^{m}$ as input, with $h_{i,0}^{m}={\tilde{x}}_{i}^{m}$. At each of the first K−1 convolutional stages, the feature is processed by a BNBlock followed by temporal max-pooling to obtain $h_{i,r}^{m}$, where r=1,…,K−1 and *K* denotes the total number of convolutional stages. Each BNBlock $B_{r}^{m}$ consists of a one-dimensional convolution, batch normalization, and a ReLU activation.

After the final convolutional stage, adaptive average pooling aggregates the remaining temporal positions to produce a fixed-dimensional epoch representation:

(10) $$f_{i}^{m}=\mathrm{AP}\left(B_{K}^{m}\left(h_{i,K-1}^{m}\right)\right),$$

where AP(·) denotes adaptive average pooling and $f_{i}^{m}\in R^{C_{f}}$ is the resulting epoch-level feature vector.

For *L* consecutive epochs, the resulting feature vectors are arranged chronologically as $F^{m}\in R^{L\times C_{f}}$, where each row corresponds to one epoch. EFE processes each epoch independently and therefore introduces no cross-epoch interaction.

### 2.6. Fused Sequence Attention (FSA)

**Figure 3:**
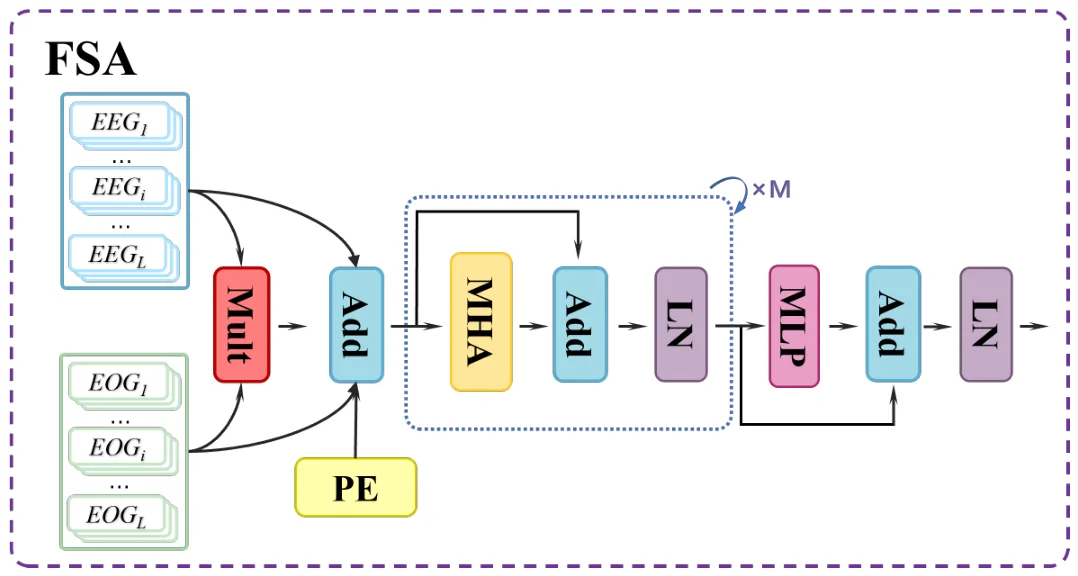
Architecture of the Fused Sequence Attention (FSA) module. The EEG and EOG epoch representations are combined through element-wise addition and multiplication. Sinusoidal positional encoding is added to the fused sequence before applying stacked multi-head self-attention layers. Each attention layer is followed by a residual connection and layer normalization. A feed-forward network with an additional residual connection and layer normalization produces the final sequence representation.

The Fused Sequence Attention (FSA) module combines the modality-specific epoch representations produced by EFE and aggregates contextual information across consecutive epochs. As illustrated in Figure 3, FSA first integrates the EEG and EOG representations through additive and element-wise interaction terms, and then applies self-attention to the resulting fused sequence.

Let $F^{\mathrm{EEG}},F^{\mathrm{EOG}}\in R^{L\times C_{f}}$ denote the EEG and EOG feature sequences output by EFE, where *L* is the number of consecutive epochs and $C_{f}$ is the epoch-feature dimension. Each row of these matrices represents one epoch in chronological order. The two modalities are fused as

(11) $$F_{fuse}=F^{\mathrm{EEG}}+F^{\mathrm{EOG}}+F^{\mathrm{EEG}}⊙F^{\mathrm{EOG}},$$

where ⊙ denotes element-wise multiplication. The additive term combines the two modality representations directly, whereas the multiplicative term introduces element-wise interactions between their corresponding feature dimensions.

To preserve the temporal order of consecutive epochs, sinusoidal positional encoding is added to the fused sequence before applying self-attention:

(12) $$F_{0}=F_{fuse}+\mathrm{PE},$$

(13) $$F_{a}={\mathrm{LN}}_{a}\left(F_{a-1}+{\mathrm{MHA}}_{a}\left(F_{a-1},F_{a-1},F_{a-1}\right)\right),$$

where a=1,…,M, and *M* is the number of self-attention layers. Because the query, key, and value are all derived from the same fused sequence, this operation performs self-attention rather than cross-attention. Each epoch representation can therefore aggregate content-dependent contextual information from the other epochs within the input sequence.

After the attention stack, a feed-forward network is applied with a residual connection and layer normalization:

(14) $$F_{\mathrm{out}}={\mathrm{LN}}_{f}\left(F_{M}+\mathrm{FFN}\left(F_{M}\right)\right),$$

where $F_{\mathrm{out}}\in R^{L\times C_{f}}$ is the resulting sequence-level representation. This representation is subsequently passed to the convolutional classification head to generate one sleep-stage prediction for each input epoch.

### 2.7. Classification and Loss Function

The sequence representation $F_{\mathrm{out}}$ is mapped to per-epoch five-class logits by a three-layer one-dimensional convolutional classification head, with a tanh activation applied after the first convolutional layer. The resulting logit matrix $Y\in R^{5\times L}$ contains one five-class logit vector for each of the *L* input epochs.

Sleep staging datasets often suffer from severe class imbalance, where the N2 stage typically contains far more samples than other stages. Under standard cross-entropy loss, optimization is dominated by majority classes, which may degrade performance on minority classes. Therefore, we adopt Focal Loss as the training objective.

Focal Loss down-weights easy examples via a modulating factor, encouraging the model to focus on hard-to-classify samples:

(15) $$\begin{array}{c} L=-\frac{1}{BL}\sum_{i=1}^{B}\sum_{j=1}^{L}\alpha_{c_{i,j}^{*}}{\left(1-p_{i,j,c_{i,j}^{*}}\right)}^{\gamma_{c_{i,j}^{*}}}\log\left(p_{i,j,c_{i,j}^{*}}\right), \end{array}$$

where *B* is the batch size, *L* is the sequence length, $p_{i,j,c_{i,j}^{*}}$ denotes the predicted probability of the ground-truth class $c_{i,j}^{*}$ for the *j*-th epoch of the *i*-th sample, $\alpha_{c_{i,j}^{*}}$ is the class weight, and $\gamma_{c_{i,j}^{*}}$ is the class-specific focusing parameter.

### 2.8. Implementation Details

RefineSleepNet was implemented in PyTorch 2.5.1 and trained on a workstation equipped with an NVIDIA GeForce RTX 4090 GPU. As described above, the input sequence length was set to 20 consecutive epochs, corresponding to a 10-min temporal context.

The AdamW optimizer was used with an initial learning rate of 0.001 and a weight decay of 0.01. The learning rate was scheduled by ReduceLROnPlateau: when the validation accuracy did not improve for five consecutive epochs, the learning rate was multiplied by 0.3. The batch size was set to 32, and the maximum number of training epochs was 60. Early stopping was applied with a patience of 15.

To address class imbalance, Focal Loss was used as the training objective. The focusing parameter was set to 2.0 for W, N2, N3, and REM, and to 3.0 for N1 to further emphasize hard N1 samples. The class weights were set to [1.0, 1.8, 1.0, 1.2, 1.25] for W, N1, N2, N3, and REM, respectively. Label smoothing was applied with ε=0.05.

The hyperparameters of Focal Loss, including the class weights, focusing parameters, and label smoothing coefficient, were determined based on the validation performance of the training set. The same configuration was applied to all datasets without dataset-specific adjustment.

Unless otherwise specified, the following architectural configuration was used in all experiments. SRU-Net adopted D=4 encoder–decoder stages with channel widths [8, 16, 32, 48, 64]. In BSAM, A=3 self-attention layers were used with 4 attention heads, where the bottleneck feature dimension was d=64 and the feed-forward network dimension was set to 4d. EFE produced $C_{f}=128$-dimensional epoch representations. In FSA, M=2 stacked multi-head self-attention layers with 8 heads were used, followed by a feed-forward network for sequence-level temporal modeling.

### 2.9. Evaluation Metrics

We evaluated cross-subject performance using subject-independent 10-fold cross-validation. In each fold, the subjects were divided into training, validation, and test sets with an approximate ratio of 8:1:1. To avoid subject leakage, all records from the same subject were assigned to the same subset. The reported metrics included overall accuracy (Acc), macro-averaged F1-score (MF1), Cohen’s kappa, and F1-score for each class.

## 3. Results

### 3.1. Experimental Results

Table 2 compares the performance of RefineSleepNet with several representative sleep-staging approaches on Sleep-EDF-39, Sleep-EDF-153, and ISRUC-3. The compared methods include conventional machine-learning approaches, CNN/RNN-based models, and attention- or Transformer-based frameworks. For the compared baselines, the reported values are obtained from their original studies or reproduced implementations under the available configurations. For reproduced baselines, we followed the training settings described in the corresponding studies whenever sufficient details were provided. When the original studies did not explicitly specify the loss function, an unweighted cross-entropy loss was adopted for training. The performance of RefineSleepNet is reported as mean ± standard deviation over ten-fold cross-validation. Since sleep-stage distributions are imbalanced, macro-F1 (MF1), class-wise F1-scores, and accuracy are reported together.

Overall, RefineSleepNet achieves competitive performance across the three datasets. It obtains MF1/Acc values of 79.8 ± 2.76%/84.5 ± 3.38%, 78.1 ± 2.34%/83.5 ± 1.74%, and 78.1 ± 3.29%/80.3 ± 3.22% on Sleep-EDF-39, Sleep-EDF-153, and ISRUC-3, respectively. Compared with conventional machine-learning approaches, deep learning-based methods generally provide better macro-F1 performance by learning discriminative representations from raw physiological signals.

Compared with CNN/RNN-based models such as DeepSleepNet, TinySleepNet, and SleepEEGNet, RefineSleepNet shows improved overall performance across the three datasets. Recent sequence-, attention-, and Transformer-based models, including SeqSleepNet, TransSleep, SleepTransformer, and CausalAttenNet, are designed to capture inter-epoch temporal dependencies and achieve competitive performance under different evaluation settings. RefineSleepNet obtains consistently higher MF1 and Acc values under the current experimental setting, indicating the effectiveness of combining signal-domain representation learning and cross-epoch temporal modeling. Paired Wilcoxon signed-rank tests were further conducted between RefineSleepNet and the strongest reproduced baseline, CausalAttenNet, based on fold-level MF1 scores. The obtained *p*-values were 0.0640, 0.0445, and 0.0755 on Sleep-EDF-39, Sleep-EDF-153, and ISRUC-3, respectively. The performance difference reached statistical significance on Sleep-EDF-153.

**Figure 4:**
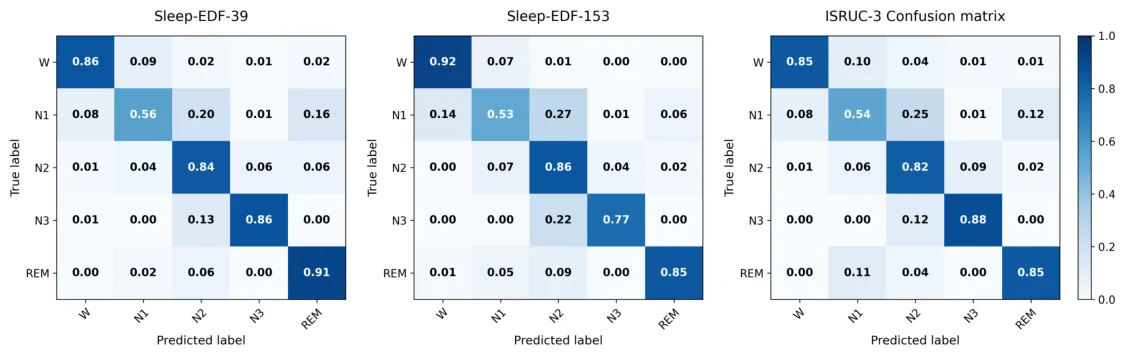
Normalized confusion matrices obtained by RefineSleepNet on Sleep-EDF-39, Sleep-EDF-153, and ISRUC-3.

For class-wise results, RefineSleepNet achieves the highest N1 F1-scores on all three datasets, with 52.2%, 52.3%, and 56.5% on Sleep-EDF-39, Sleep-EDF-153, and ISRUC-3, respectively. As shown in the normalized confusion matrices in Figure 4, Wake, N2, N3, and REM are generally well recognized, whereas N1 is still mainly confused with Wake, N2, and REM. Although RefineSleepNet achieves comparatively higher N1 F1-scores, N1 remains its most challenging sleep stage.

In terms of model size, RefineSleepNet contains approximately 0.8 M trainable parameters.

### 3.2. Ablation Experiment

#### 3.2.1. Component-Wise Ablation

To evaluate the contribution of each component within RefineSleepNet, we designed the following variants:

- **Add-Only:** removes the multiplicative interaction term and the sequence-level self-attention module, retaining only additive EEG/EOG fusion.
- **Fusion-Only:** retains additive and multiplicative fusion operations but removes the sequence-level self-attention module.
- **Add-only + FSA:** removes the multiplicative fusion branch while retaining additive fusion and FSA.
- **Multiply-only + FSA:** removes the additive fusion branch while retaining multiplicative fusion and FSA.
- **Concat fusion:** replaces the proposed interaction-based fusion with feature concatenation.
- **SRU-Net w/o BSAM:** removes the bottleneck self-attention module while preserving the remaining SRU-Net architecture.
- **w/o α:** removes the learnable residual connection coefficient in SRU-Net.
- **CNN control:** replaces SRU-Net with a CNN-based encoder-decoder while maintaining a comparable number of trainable parameters.
- **FSA + w/o PE:** removes positional encoding from the sequence-level self-attention module.
- **EEG-only:** removes the EOG branch and performs sleep staging using EEG representations only.

As shown in Table 3, the full RefineSleepNet achieves the highest MF1 among all tested variants on the three datasets, with scores of 79.8, 78.1, and 78.1 on Sleep-EDF-39, Sleep-EDF-153, and ISRUC-3, respectively, indicating that the proposed components provide complementary improvements when combined.

Regarding multimodal fusion, Add-Only and Fusion-Only evaluate the influence of fusion operations without sequence-level temporal modeling. Compared with the complete model, both variants show performance degradation, indicating that cross-epoch contextual modeling is important after multimodal fusion.

To further investigate the impact of different feature fusion strategies, we evaluated two variants, Add-only + FSA and Multiply-only + FSA, where the multi-head self-attention mechanism in FSA was retained while replacing the original fusion operation with addition-only or multiplication-only fusion. The results show that neither additive nor multiplicative interaction alone can achieve the performance of the complete fusion strategy, suggesting that complementary information is captured through the combination of both interaction mechanisms. Concat fusion obtains lower MF1 than the proposed fusion strategy on the evaluated datasets, indicating that explicitly modeling EEG/EOG interactions is beneficial for multimodal representation learning. For signal-domain representation learning, the CNN control variant was designed by replacing SRU-Net with a CNN-based encoder–decoder while maintaining a comparable parameter scale. Compared with CNN control, RefineSleepNet achieves higher MF1 performance, suggesting that the proposed SRU-Net provides more effective signal-domain representations under similar model capacity. Furthermore, removing BSAM decreases MF1 from 79.8% to 76.6% on Sleep-EDF-39, from 78.1% to 77.3% on Sleep-EDF-153, and from 78.1% to 76.4% on ISRUC-3. The w/o α variant further evaluates the contribution of the learnable residual connection in SRU-Net. The performance variation after removing this adaptive residual coefficient suggests that the residual pathway contributes to signal-domain representation learning.

For sequence-level modeling, FSA + w/o PE produces lower performance than the full model, indicating that positional information contributes to modeling directional transitions across consecutive sleep epochs. For modality contribution, EEG-only shows reduced performance compared with the full model, demonstrating that EOG provides complementary information for sleep-stage discrimination.

#### 3.2.2. Effect of Loss Function and Signal Processing Strategy

To investigate the influence of the training objective and front-end design, we further conducted two additional comparisons based on the complete RefineSleepNet. First, the original Focal Loss was replaced with a plain cross entropy (CE) loss while keeping the network architecture unchanged. Second, the proposed SRU-Net front-end was replaced with a fixed bandpass filtering strategy to evaluate the contribution of the learnable signal-domain representation. The results are summarized in Table 4.

Compared with plain CE loss, the original Focal Loss configuration achieves higher MF1 and Kappa values across all three datasets. The improvement is consistent on both Sleep-EDF and ISRUC-3, indicating that the adopted loss function provides additional optimization benefits under the imbalanced distribution of sleep stages.

Compared with the Fixed Bandpass variant, the complete RefineSleepNet achieves higher MF1 performance on all datasets. The MF1 improvements are 3.6, 3.2, and 7.3 percentage points on Sleep-EDF-39, Sleep-EDF-153, and ISRUC-3, respectively. These results indicate that replacing manually designed filtering with the learnable SRU-Net front-end provides more adaptive signal representations for subsequent feature extraction and sequence-level modeling.

### 3.3. Signal-Domain Transformation Analysis

To understand how SRU-Net modifies the input signals under the sleep staging objective, we analyzed the SRU-Net outputs from three complementary perspectives: time-domain consistency and signal transformation, intra-epoch attention responses, and frequency-domain spectral modulation. The purpose of this analysis was not to treat SRU-Net as a conventional signal restoration module or to verify input replication. Instead, we examined whether the SRU-Net outputs before epoch-level feature extraction retain broad temporal and spectral structures of the inputs while exhibiting task-driven signal-domain transformations.

#### 3.3.1. Time-Domain Consistency and Signal Transformation

**Figure 5:**
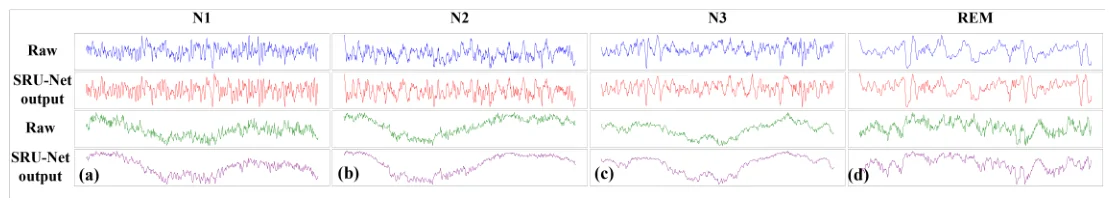
Time-domain examples of raw EEG/EOG inputs and the corresponding SRU-Net outputs for different sleep stages: (**a**) N1; (**b**) N2; (**c**) N3; and (**d**) REM. The examples illustrate broad input–output structural similarity together with local waveform modifications.

The time-domain examples in Figure 5 show that the SRU-Net outputs generally preserve the overall waveform morphology and temporal trends of the raw EEG and EOG inputs, while changes can be observed in local amplitudes and fine-scale waveform details. These qualitative observations indicate that the SRU-Net outputs maintain the essential temporal structures of physiological signals while performing task-driven signal-domain transformation for sleep-stage discrimination.

To quantitatively characterize the relationship between the raw inputs and the transformed outputs, Pearson correlation coefficient, mean squared error (MSE), mean absolute error (MAE), and Relative L2 change were calculated between the input signals and SRU-Net outputs. The results are summarized in Table 5. The Pearson correlations are 0.839 ± 0.061 for EEG and 0.792 ± 0.183 for EOG, indicating that the transformed signals maintain considerable similarity with the original physiological signals. Meanwhile, the non-zero MSE, MAE, and Relative L2 values indicate that SRU-Net introduces signal modifications rather than directly reproducing the input signals.

Overall, these results suggest that SRU-Net preserves the dominant temporal organization of EEG/EOG signals while modifying waveform characteristics relevant to sleep-stage classification.

#### 3.3.2. Intra-Epoch Attention Responses

**Figure 6:**
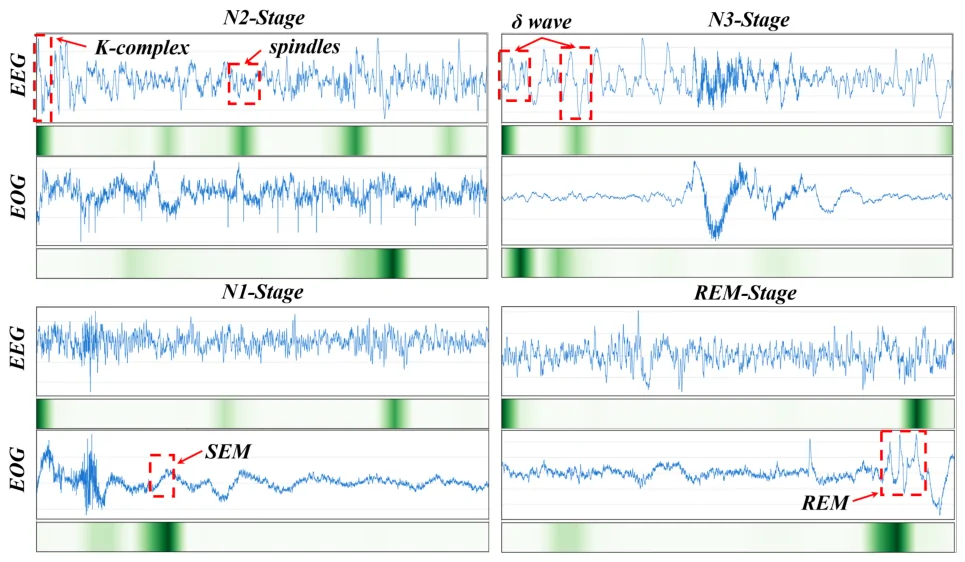
Qualitative intra-epoch BSAM attention responses extracted from the bottleneck self-attention module across different sleep stages. The attention maps illustrate non-uniform temporal weighting over intra-epoch representations, where the color intensity represents the relative attention response at different temporal positions during signal-domain transformation.

Figure 6 shows the intra-epoch BSAM response patterns extracted from the bottleneck self-attention module across different sleep stages. The response maps exhibit non-uniform distributions across temporal positions within each epoch, indicating that BSAM assigns different levels of attention to different temporal regions. Notably, relatively high attention responses are observed around locally distinctive waveform regions in EEG and EOG signals. These qualitative observations suggest that BSAM adaptively emphasizes temporally informative patterns during signal-domain transformation.

#### 3.3.3. Frequency-Domain Spectral Modulation

**Figure 7:**
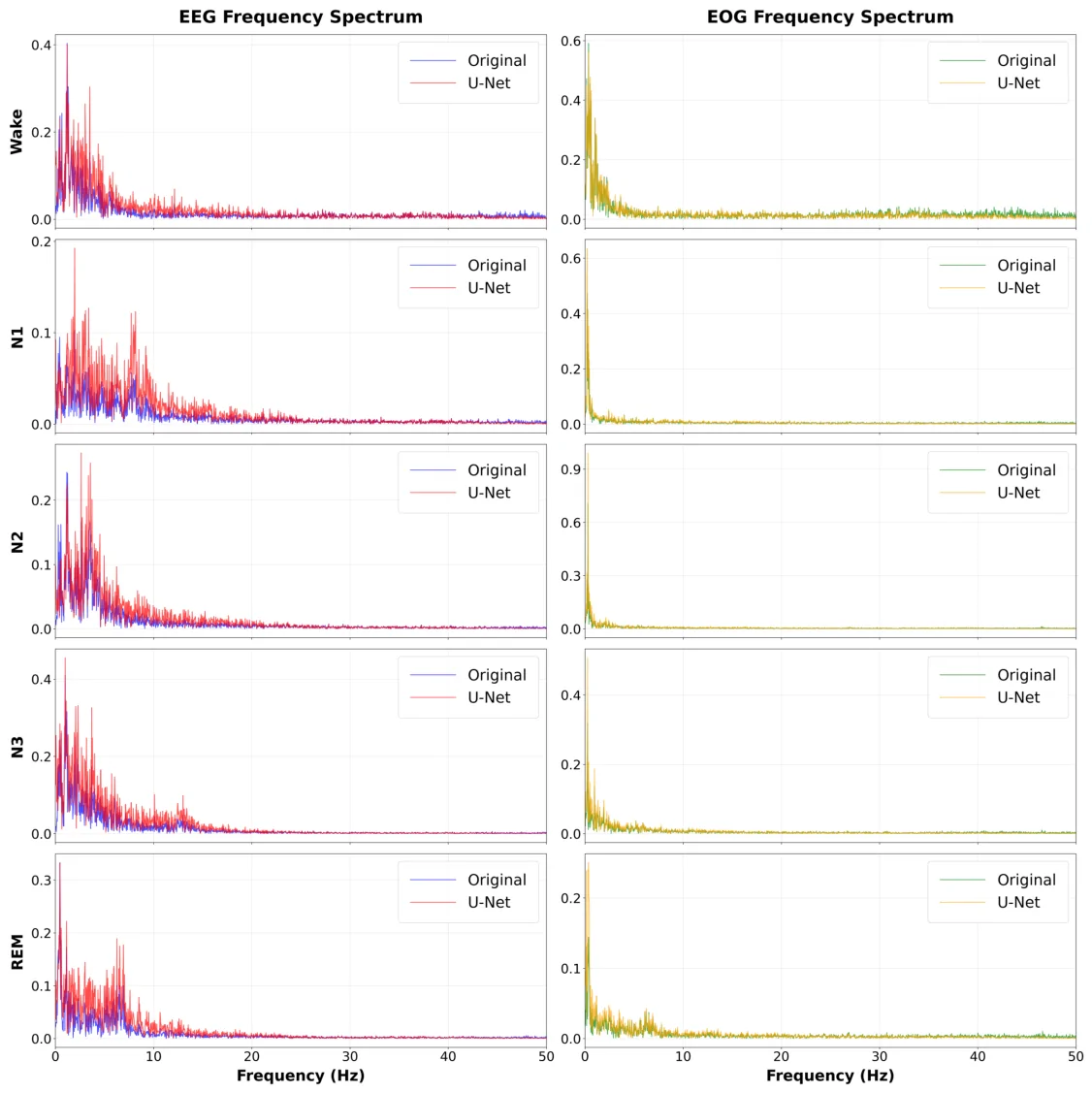
FFT amplitude spectra of the raw EEG/EOG inputs and the corresponding SRU-Net outputs for N1, N2, N3, and REM.

Figure 7 compares the FFT amplitude spectra of representative 30 s raw EEG/EOG inputs and their corresponding SRU-Net outputs. The dominant spectral profiles and major peak locations of the raw inputs remain broadly visible in the corresponding SRU-Net outputs. For EEG, the SRU-Net outputs exhibit spectral modulation mainly in low-frequency bands, whereas the differences at higher frequencies are relatively limited or show attenuation. The EOG spectra exhibit more moderate changes that are mainly concentrated in the lower-frequency range.

**Figure 8:**
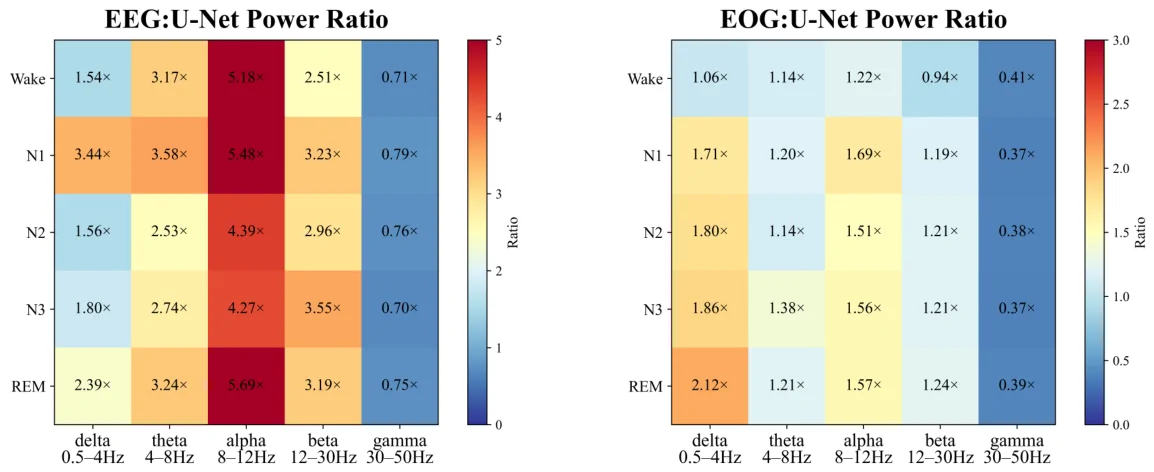
Band-power ratios between the SRU-Net outputs and raw inputs for N1, N2, N3, and REM. The ratio is defined as the output band power divided by the input band power.

To further characterize these changes, Figure 8 presents output-to-input band-power ratios calculated from the power spectral density estimates obtained using Welch’s method over delta (0.5–4 Hz), theta (4–8 Hz), alpha (8–12 Hz), beta (12–30 Hz), and gamma (30–50 Hz). For EEG, the output-to-input ratios indicate frequency-dependent redistribution, with relatively increased power mainly observed in low-frequency bands and reduced power in the gamma band. EOG shows more moderate spectral redistribution together with a consistent reduction in gamma-band power.

Overall, the FFT spectra and band-power ratios indicate that SRU-Net preserves the broad spectral organization of the input signals while introducing frequency-selective and modality-specific spectral modulation. These observations suggest that SRU-Net performs adaptive signal transformation rather than applying a fixed frequency filtering strategy.

### 3.4. Sleep Architecture Analysis

**Figure 9:**
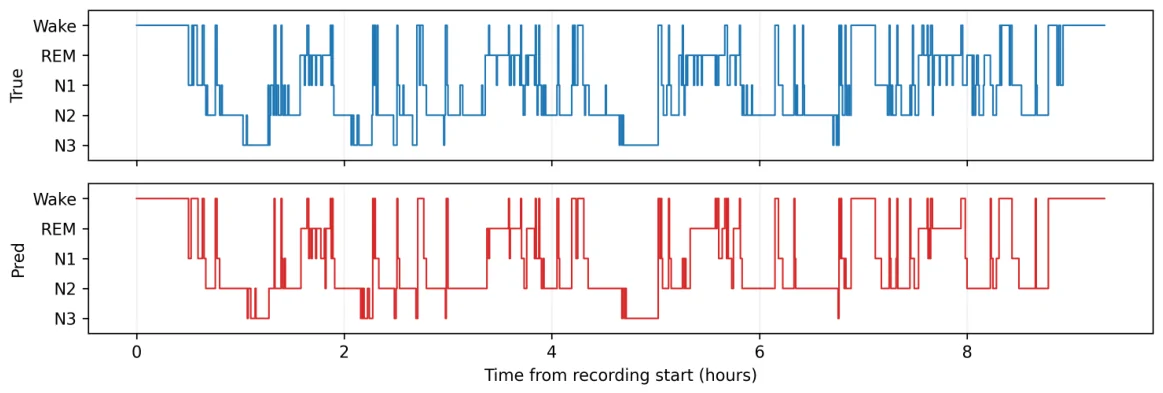
Sleep-window hypnogram comparison for record SC4291G0. The upper panel shows the ground-truth hypnogram, and the lower panel shows the predicted hypnogram.

To evaluate whether the predicted hypnograms preserve the temporal organization of overnight sleep, we further analyzed global sleep statistics, stage composition, and temporal characteristics across all test subjects. Figure 9 shows an example sleep-window hypnogram comparison between the reference annotation and RefineSleepNet prediction. The predicted hypnogram generally follows the overall sleep-stage progression of the reference signal, while some short-duration stage transitions are not fully retained. The quantitative comparison across all test subjects is summarized in Table 6.

**Figure 10:**
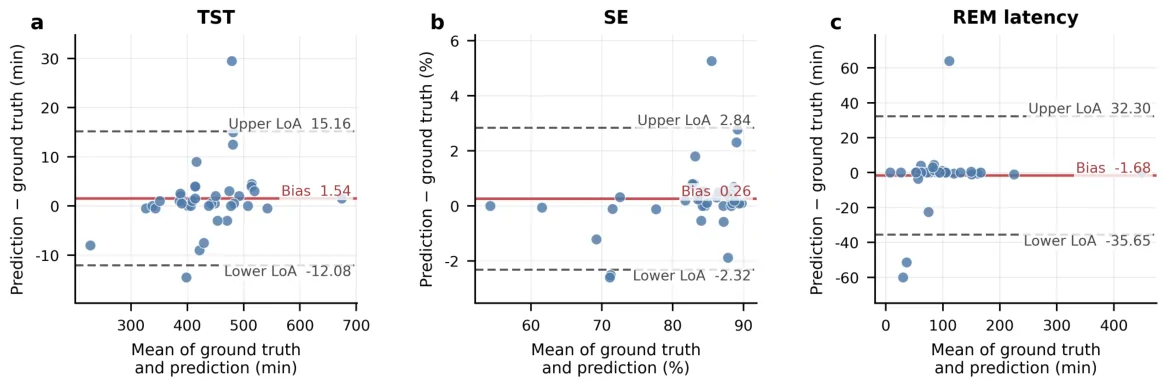
Bland–Altman analyses of predicted sleep architecture parameters: (**a**) total sleep time (TST); (**b**) sleep efficiency (SE); and (**c**) REM latency.

RefineSleepNet shows close agreement with the reference annotations for several global sleep parameters, particularly TST and SE. The mean signed errors of TST, SE, and REM latency are +1.54 min, +0.26%, and −1.68 min, respectively, indicating limited systematic bias in these global sleep parameters. In contrast, the number of sleep-stage transitions is underestimated, with 111.87 ± 28.52 transitions in the reference annotations and 96.39 ± 26.66 in the predictions, corresponding to a mean signed error of −15.47 ± 18.84. This discrepancy indicates that the predicted hypnograms tend to be smoother and may not fully preserve brief stage changes or fragmented sleep structures. Bland–Altman analyses were further performed for TST, SE, and REM latency, as shown in Figure 10. For TST, the bias was 1.54 min with limits of agreement from −12.08 to 15.16 min. For SE, the bias was 0.26% with limits of agreement from −2.32% to 2.84%. For REM latency, the bias was −1.68 min with limits of agreement from −35.65 to 32.30 min. Overall, these results indicate that RefineSleepNet preserves several major global sleep architecture characteristics beyond epoch-level classification performance, while transition-related temporal details remain less accurately reproduced.

## 4. Discussion

The results across Sleep-EDF-39, Sleep-EDF-153, and ISRUC-3 support the effectiveness of the hierarchical modeling strategy adopted in RefineSleepNet. By introducing a modality-specific and sample-aligned signal-domain transformation before epoch-level feature compression, the model provides an explicit intermediate representation stage before subsequent multimodal integration and sequence-level contextual modeling. The consistent performance of the complete model, together with the ablation results, supports the contribution of this staged organization. In particular, the comparisons with the shallow convolutional front end and the model without sequence-level attention indicate that the complete SRU-Net front end and subsequent temporal contextualization both contribute to the final staging performance. Taken together, these findings highlight the value of coordinating signal-domain, epoch-level, and sequence-level representation learning within a unified framework.

The representation analyses further clarify the role of the signal-domain transformation. The SRU-Net outputs retained the broad temporal and spectral organization of the input signals while exhibiting local waveform modifications, non-uniform intra-epoch responses, and modality-specific spectral redistribution. These complementary observations support the interpretation that SRU-Net learns a task-driven intermediate waveform representation rather than simply reproducing the raw input. In the illustrative full-record case, the predicted hypnogram broadly followed the overnight sleep progression and showed relatively small deviations in total sleep time, sleep efficiency, and REM latency, although larger differences remained in stage composition. The reduced number of predicted stage transitions indicates that RefineSleepNet tends to generate smoother hypnograms, which may lead to underestimation of highly fragmented sleep patterns and short-duration stage fluctuations. This limitation suggests that further modeling of transition dynamics and event-level temporal variability may be beneficial for improving fine-grained sleep architecture reconstruction.

Despite the overall improvements, several limitations remain. First, although RefineSleepNet improves N1 recognition and overall macro-F1 performance, N1 remains a challenging stage due to its transitional characteristics and overlap with neighboring sleep stages. Second, the current sequence-level modeling focuses on contextual dependencies among consecutive epochs but does not explicitly model sleep-stage transition probabilities or physiological event timing. Therefore, future work could incorporate transition-aware objectives or event-level constraints to better preserve fragmented sleep structures. Finally, the current representation analysis mainly evaluates signal transformations and sleep architecture characteristics; broader validation on larger and more diverse cohorts will be necessary to further assess the generalizability of the learned representations.

## 5. Conclusions

In this paper, we propose RefineSleepNet, a hierarchical framework for automatic sleep staging from EEG/EOG recordings. Its central design explicitly separates modality-specific, sample-aligned, and task-driven signal-domain transformation from subsequent epoch-level feature extraction, while coordinating multimodal integration and cross-epoch contextual modeling. Results across three public datasets demonstrate the effectiveness of the proposed framework, while the ablation experiments provide component-level evidence for the contribution of the proposed modules. Complementary analyses of the intermediate waveform representations further indicate that the learned transformation retains broad temporal and spectral organization while introducing non-uniform temporal and spectral modulation rather than simply reproducing the input signals. Additional statistical analysis and sleep architecture evaluation further support the reliability of the obtained sleep staging results. Future work will investigate subject- and context-adaptive signal-domain transformation and transition-aware contextual modeling to better represent boundary epochs, fragmented sleep patterns, and brief stage transitions. 

## Figures and Tables

**Table 1:** Summary of the datasets.

Dataset	Wake	N1	N2	N3	REM
Sleep-EDF-39	8285	2804	17,799	5703	7717
Sleep-EDF-153	65,951	21,522	69,132	13,039	25,835
ISRUC-3	1713	1248	2678	2035	1111

**Table 2:** Performance comparison of the approaches on three datasets.

Dataset	Method	Overall Result			F1-Score for Each Class				
		MF1	Acc	Kappa	Wake	N1	N2	N3	REM
Sleep-EDF-39	SVM [29]	73.3	78.3	71.0	82.0	40.7	84.0	82.9	76.9
	RF [30]	72.3	80.6	73.5	81.6	34.7	85.6	83.1	76.7
	DeepSleepNet [8]	76.9	83.3	76.8	89.2	42.5	86.5	83.1	82.2
	TinySleepNet [9]	73.1	79.0	71.4	81.3	44.5	81.6	77.4	80.8
	SleepEEGNet [10]	76.9	83.2	76.7	**89.2**	45.3	86.1	83.2	80.8
	XSleepNet [23]	77.5	83.7	77.4	87.4	46.2	86.8	83.3	83.7
	SeqSleepNet [11]	77.5	83.4	77.1	88.9	46.5	86.6	84.3	81.5
	TransSleep [17]	73.5	81.7	74.1	82.5	34.5	85.9	84.1	80.4
	SleepTransformer [16]	76.6	83.1	76.6	87.0	44.8	86.2	81.6	83.3
	CausalAttenNet [18]	77.0	83.2	76.8	87.8	43.8	**87.0**	**84.8**	81.5
	**RefineSleepNet**	**79.8**	**84.5**	**78.6**	88.8	**52.2**	86.5	84.1	**87.1**
		±2.76	±3.38	±4.66	±3.90	±5.10	±4.34	±5.58	±5.60
Sleep-EDF-153	SVM [29]	70.4	74.6	66.2	87.1	45.1	77.4	70.6	71.9
	RF [30]	68.6	77.5	68.3	86.8	31.1	82.0	72.8	70.6
	DeepSleepNet [8]	74.0	81.2	73.9	91.7	44.6	83.9	69.2	80.8
	TinySleepNet [9]	74.1	79.6	72.0	89.5	47.8	81.5	70.8	80.6
	SleepEEGNet [10]	75.4	82.5	75.6	92.4	48.1	84.5	70.6	81.5
	XSleepNet [23]	75.8	82.5	75.6	92.2	48.4	84.6	72.1	81.1
	SeqSleepNet [11]	74.5	81.2	74.0	92.1	47.1	83.2	72.1	77.9
	TransSleep [17]	74.9	81.2	74.3	91.9	46.0	83.7	71.1	81.9
	SleepTransformer [16]	73.9	81.5	74.1	91.8	43.8	83.9	68.6	81.6
	CausalAttenNet [18]	75.2	82.1	75.7	92.1	46.1	84.2	73.0	80.5
	**RefineSleepNet**	**78.1**	**83.5**	**77.1**	**92.6**	**52.3**	**84.7**	**75.0**	**86.0**
		±2.34	±1.74	±2.48	±2.63	±3.79	±1.71	±6.70	±3.66
ISRUC-3	SVM [29]	70.8	73.4	65.3	80.3	47.5	71.5	84.6	70.3
	RF [30]	70.1	74.1	65.8	80.5	44.6	74.8	84.5	66.3
	DeepSleepNet [8]	70.1	72.4	64.5	77.8	48.7	66.5	86.9	70.5
	TinySleepNet [9]	60.7	63.6	53.8	59.4	43.7	58.7	75.2	66.6
	SleepEEGNet [10]	66.5	70.5	61.7	79.0	44.7	68.3	74.5	66.0
	XSleepNet [23]	71.7	75.8	68.1	82.0	47.2	74.5	84.8	70.1
	SeqSleepNet [11]	69.4	74.1	66.0	81.2	44.4	73.4	86.2	61.7
	TransSleep [17]	67.8	72.9	64.3	77.3	42.1	74.2	86.3	59.1
	SleepTransformer [16]	67.2	70.7	62.6	82.5	48.4	67.0	78.1	59.9
	CausalAttenNet [18]	73.9	77.4	70.2	83.1	50.1	76.0	86.0	74.1
	**RefineSleepNet**	**78.1**	**80.3**	**75.7**	**85.0**	**56.5**	**78.5**	**87.5**	**83.0**
		±3.29	±3.22	±4.94	±3.76	±6.24	±4.45	±3.75	±5.22

The bold result and underlined result are the best result and the second best result, respectively.

**Table 3:** Ablation study of RefineSleepNet on three datasets. Each entry represents MF1/Acc/Kappa.

Variant	Sleep-EDF-39	Sleep-EDF-153	ISRUC-3
RefineSleepNet	79.8/84.5/78.6	78.1/83.5/77.1	78.1/80.3/75.7
*Multimodal fusion*			
Add-Only	75.6/81.8/75.9	75.9/82.1/75.2	69.8/74.0/65.8
Fusion-Only	74.1/81.1/73.6	75.0/81.6/74.5	66.0/71.8/62.7
Add-only + FSA	78.1/83.3/77.2	77.0/82.9/77.0	75.4/78.4/72.7
Multiply-only + FSA	77.2/83.2/76.5	76.7/82.4/76.3	74.3/77.9/72.1
Concat fusion	76.3/82.3/75.3	76.7/82.6/75.9	76.5/81.7/74.3
*Signal-domain transformation*			
SRU-Net w/o BSAM	76.6/82.1/75.3	77.3/83.1/76.7	76.4/79.3/72.8
w/o α	78.2/83.6/77.2	76.7/83.2/76.6	77.1/79.1/74.3
CNN control	78.2/83.9/77.8	76.8/82.5/75.9	75.9/78.5/72.7
*Sequence modeling and modality*			
FSA + w/o PE	78.7/83.9/77.9	77.1/82.8/76.2	77.1/79.2/74.6
EEG-only	77.2/83.2/76.8	76.1/82.1/75.2	75.3/78.5/73.2

**Table 4:** Effect of training objective and front-end design. Each entry represents MF1/Acc/Kappa.

Dataset	Variant	MF1 (%)	Acc (%)	Kappa
Sleep-EDF-39	Full (Focal Loss)	79.8	84.5	78.6
	CE	78.3	84.2	77.9
	Fixed Bandpass	76.2	83.2	76.8
Sleep-EDF-153	Full (Focal Loss)	78.1	83.5	77.1
	CE	77.2	82.7	75.8
	Fixed Bandpass	74.9	82.8	75.9
ISRUC-3	Full (Focal Loss)	78.1	80.3	75.7
	CE	77.1	80.1	73.7
	Fixed Bandpass	70.8	74.8	66.9

**Table 5:** Input–output consistency between raw signals and SRU-Net transformed signals.

Modality	Pearson	MSE	MAE	Relative L2
EEG	0.839±0.061	0.429±0.186	0.432±0.138	0.748±0.051
EOG	0.792±0.183	0.384±0.103	0.388±0.174	0.781±0.157

**Table 6:** Comparison of predicted and reference sleep architecture parameters across all test subjects.

Metric	Ground Truth	Prediction	Signed Error
TST (min)	436.18 ± 73.46	437.72 ± 75.46	+1.54 ± 6.95
SE (%)	82.31 ± 7.98	82.57 ± 8.41	+0.26 ± 1.31
REM latency (min)	96.34 ± 73.37	94.66 ± 75.06	−1.68 ± 17.34
N1% of TST	8.26 ± 4.71	7.54 ± 4.68	−0.72 ± 2.10
N2% of TST	51.57 ± 9.24	50.80 ± 7.49	−0.77 ± 3.34
N3% of TST	17.46 ± 9.58	18.90 ± 8.06	+1.44 ± 3.06
REM% of TST	22.72 ± 5.56	22.76 ± 5.53	+0.05 ± 1.21
Transitions	111.87 ± 28.52	96.39 ± 26.66	−15.47 ± 18.84
REM episodes	10.13 ± 6.72	9.18 ± 5.19	−0.95 ± 3.67

## Data Availability

The datasets analyzed in this study are publicly available. Sleep-EDF-39 and Sleep-EDF-153 were obtained from the Sleep-EDF Database Expanded available through PhysioNet, and ISRUC-3 was obtained from Subgroup III of the ISRUC-Sleep dataset.

## References

[1] McNaughton B.L. (2010). Cortical Hierarchies, Sleep, and the Extraction of Knowledge from Memory. Artif. Intell..

[2] Iber C., Ancoli-Israel S., Chesson A.L., Quan S.F. (2007). The AASM Manual for the Scoring of Sleep and Associated Events: Rules, Terminology and Technical Specifications.

[3] Aboalayon K.A.I., Faezipour M., Almuhammadi W.S., Moslehpour S. (2016). Sleep Stage Classification Using EEG Signal Analysis: A Comprehensive Survey and New Investigation. Entropy.

[4] Phan H., Andreotti F., Cooray N., Chén O.Y., De Vos M. (2019). Joint Classification and Prediction CNN Framework for Automatic Sleep Stage Classification. IEEE Trans. Biomed. Eng..

[5] Sokolovsky M., Guerrero F., Paisarnsrisomsuk S., Ruiz C., Alvarez S.A. (2020). Deep Learning for Automated Feature Discovery and Classification of Sleep Stages. IEEE/ACM Trans. Comput. Biol. Bioinform..

[6] An P., Zhao J., Du B., Zhao W., Zhang T., Yuan Z. (2024). Amplitude–Time Dual-View Fused EEG Temporal Feature Learning for Automatic Sleep Staging. IEEE Trans. Neural Netw. Learn. Syst..

[7] Perslev M., Jensen M., Darkner S., Jennum P.J., Igel C., Wallach H., Larochelle H., Beygelzimer A., d’Alché-Buc F., Fox E., Garnett R. (2019). U-Time: A Fully Convolutional Network for Time Series Segmentation Applied to Sleep Staging. Advances in Neural Information Processing Systems 32.

[8] Supratak A., Dong H., Wu C., Guo Y. (2017). DeepSleepNet: A Model for Automatic Sleep Stage Scoring Based on Raw Single-Channel EEG. IEEE Trans. Neural Syst. Rehabil. Eng..

[9] Supratak A., Guo Y. (2020). TinySleepNet: An Efficient Deep Learning Model for Sleep Stage Scoring Based on Raw Single-Channel EEG. Proceedings of the 2020 42nd Annual International Conference of the IEEE Engineering in Medicine & Biology Society (EMBC).

[10] Mousavi S., Afghah F., Acharya U.R. (2019). SleepEEGNet: Automated Sleep Stage Scoring with Sequence to Sequence Deep Learning Approach. PLoS ONE.

[11] Phan H., Andreotti F., Cooray N., Chén O.Y., De Vos M. (2019). SeqSleepNet: End-to-End Hierarchical Recurrent Neural Network for Sequence-to-Sequence Automatic Sleep Staging. IEEE Trans. Neural Syst. Rehabil. Eng..

[12] Phan H., Lorenzen K.P., Heremans E., Chén O.Y., Tran M.C., Koch P., Mertins A., Baumert M., Mikkelsen K.B., De Vos M. (2023). L-SeqSleepNet: Whole-cycle Long Sequence Modeling for Automatic Sleep Staging. IEEE J. Biomed. Health Inform..

[13] Phan H., Andreotti F., Cooray N., Chén O.Y., Vos M.D. (2018). Automatic Sleep Stage Classification Using Single-Channel EEG: Learning Sequential Features with Attention-Based Recurrent Neural Networks. Proceedings of the 2018 40th Annual International Conference of the IEEE Engineering in Medicine and Biology Society (EMBC).

[14] Eldele E., Chen Z., Liu C., Wu M., Kwoh C.K., Li X., Guan C. (2021). An Attention-Based Deep Learning Approach for Sleep Stage Classification With Single-Channel EEG. IEEE Trans. Neural Syst. Rehabil. Eng..

[15] Seo H., Back S., Lee S., Park D., Kim T., Lee K. (2020). Intra- and Inter-Epoch Temporal Context Network (IITNet) Using Sub-Epoch Features for Automatic Sleep Scoring on Raw Single-Channel EEG. Biomed. Signal Process. Control.

[16] Phan H., Mikkelsen K., Chén O.Y., Koch P., Mertins A., De Vos M. (2022). SleepTransformer: Automatic Sleep Staging With Interpretability and Uncertainty Quantification. IEEE Trans. Biomed. Eng..

[17] Phyo J., Ko W., Jeon E., Suk H.I. (2023). TransSleep: Transitioning-Aware Attention-Based Deep Neural Network for Sleep Staging. IEEE Trans. Cybern..

[18] Pan J., Feng Y., Zhao P., Zou X., Hou A., Che X. (2024). CausalAttenNet: A Fast and Long-Term-Temporal Network for Automatic Sleep Staging With Single-Channel EEG. IEEE Trans. Instrum. Meas..

[19] Liu C., Guan Q., Zhang W., Sun L., Wang M., Dong X., Xu S. (2025). MultiScaleSleepNet: A Hybrid CNN–BiLSTM–Transformer Architecture with Multi-Scale Feature Representation for Single-Channel EEG Sleep Stage Classification. Sensors.

[20] Chambon S., Galtier M.N., Arnal P.J., Wainrib G., Gramfort A. (2018). A Deep Learning Architecture for Temporal Sleep Stage Classification Using Multivariate and Multimodal Time Series. IEEE Trans. Neural Syst. Rehabil. Eng..

[21] Wang I.N., Lee C.H., Kim H.J., Kim H., Kim D.J. (2020). An Ensemble Deep Learning Approach for Sleep Stage Classification via Single-channel EEG and EOG. Proceedings of the 2020 International Conference on Information and Communication Technology Convergence (ICTC).

[22] Samaee M., Yazdi M., Massicotte D. (2025). NeuroLingua: A Language-Inspired Hierarchical Framework for Multimodal Sleep Stage Classification Using EEG and EOG. arXiv.

[23] Phan H., Chén O.Y., Tran M.C., Koch P., Mertins A., De Vos M. (2022). XSleepNet: Multi-View Sequential Model for Automatic Sleep Staging. IEEE Trans. Pattern Anal. Mach. Intell..

[24] Phan H., Chén O.Y., Koch P., Mertins A., De Vos M. (2019). Fusion of End-to-End Deep Learning Models for Sequence-to-Sequence Sleep Staging. Proceedings of the 2019 41st Annual International Conference of the IEEE Engineering in Medicine and Biology Society (EMBC).

[25] Goldberger A.L., Amaral L.A., Glass L., Hausdorff J.M., Ivanov P.C., Mark R.G., Mietus J.E., Moody G.B., Peng C.K., Stanley H.E. (2000). PhysioBank, PhysioToolkit, and PhysioNet: Components of a New Research Resource for Complex Physiologic Signals. Circulation.

[26] Kemp B., Zwinderman A., Tuk B., Kamphuisen H., Oberye J. (2000). Analysis of a Sleep-Dependent Neuronal Feedback Loop: The Slow-Wave Microcontinuity of the EEG. IEEE Trans. Biomed. Eng..

[27] Khalighi S., Sousa T., Santos J.M., Nunes U. (2016). ISRUC-Sleep: A Comprehensive Public Dataset for Sleep Researchers. Comput. Methods Programs Biomed..

[28] Imtiaz S.A., Rodriguez-Villegas E. (2014). Recommendations for Performance Assessment of Automatic Sleep Staging Algorithms. Proceedings of the 2014 36th Annual International Conference of the IEEE Engineering in Medicine and Biology Society.

[29] Hearst M., Dumais S., Osuna E., Platt J., Scholkopf B. (1998). Support Vector Machines. IEEE Intell. Syst..

[30] Breiman L. (2001). Random Forests. Mach. Learn..

